# Association Between Seasonal Influenza and Absolute Humidity: Time-Series Analysis with Daily Surveillance Data in Japan

**DOI:** 10.1038/s41598-020-63712-2

**Published:** 2020-05-08

**Authors:** Keita Shimmei, Takahiro Nakamura, Chris Fook Sheng Ng, Masahiro Hashizume, Yoshitaka Murakami, Aya Maruyama, Takako Misaki, Nobuhiko Okabe, Yuji Nishiwaki

**Affiliations:** 1 The Poverty and Equity Global Practice, The World Bank, Washington, DC US; 2 Department of Environmental and Occupational Health, Toho University, Tokyo, Japan; 3 School of Tropical Medicine and Global Health, Nagasaki University, Nagasaki, Japan; 4 Department of Global Health Policy, Graduate School of Medicine, The University of Tokyo, Tokyo, Japan; 5 Department of Medical Statistics, Toho University, Tokyo, Japan; 6 Kawasaki City Institute for Public Health, Kawasaki, Japan

**Keywords:** Influenza virus, Epidemiology

## Abstract

Seasonal influenza epidemics are associated with various meteorological factors. Recently absolute humidity (AH) has garnered attention, and some epidemiological studies show an association between AH and human influenza infection. However, they mainly analyzed weekly surveillance data, and daily data remains largely unexplored despite its potential benefits. In this study, we analyze daily influenza surveillance data using a distributed lag non-linear model to examine the association of AH with the number of influenza cases and the magnitude of the association. Additionally, we investigate how adjustment for seasonality and autocorrelation in the model affect results. All models used in the study showed a significant increase in the number of influenza cases as AH decreased, although the magnitude of the association differed substantially by model. Furthermore, we found that relative risk reached a peak at lag 10–14 with extremely low AH. To verify these findings, further analysis should be conducted using data from other locations.

## Introduction

Seasonal influenza epidemics are a serious public health concern, as they are associated with increased hospital admissions and an estimated 291,000–646,000 seasonal influenza-associated respiratory deaths annually worldwide^1^. Influenza epidemics are also an economic problem-financial losses attributable to influenza in the United States amounted to around US $90 billion in^2^. It is therefore essential to identify factors that contribute to the development of influenza epidemics.

During the past few decades, studies have examined associations of influenza epidemics with meteorological factors^3–11^. Recently, the effect of absolute humidity (AH; i.e. the density of water mass in air [g/m^3^]) on the survival of influenza virus has been gained attention. Evidence from laboratory experiments suggest that influenza virus survival is more closely associated with AH than with temperature or relative humidity^12,13^. Additionally, a few epidemiological studies, which analyzed surveillance data, shows negative association of AH with influenza infection^14–17^. However, these studies mainly investigated weekly surveillance data. Analysis with more granular data, such as daily data, has obvious possibility to bring accurate estimates in the association, but remain largely unexplored.

In such studies investigating associations of infectious diseases with meteorological factors, time-series analysis is widely used^18–29^. This type of analysis must address two issues: control for autocorrelation (i.e. how to account for the effect of previously infected people) and control for seasonality. Although the approaches selected to address these problems greatly affect study findings, few studies have examined discrepancies in results obtained with differing methods.

In this study, we conducted time-series analysis of daily influenza surveillance data to examine the association of AH with the number of influenza cases and the strength of associations. In addition, this study examined how adjustment for seasonality and autocorrelation affected the results of the analysis.

## Results

**Figure 1:**
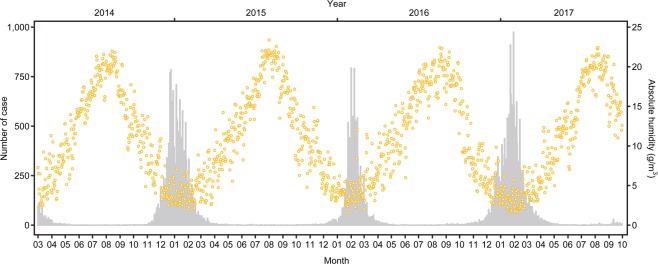
Epidemic curve (grey bar) and daily AH (yellow plot) from March 2014 to October 2017. N = 1310 days.

To identify any trend in AH and influenza cases, we graphed the epidemic curve and plotted daily AH during the entire study period, from March 2014 through October 2017 (Fig. 1). Three obvious influenza epidemics were revealed during this period. The number of influenza cases usually started to increase in November or December, reached a peak in January or February, and decreased to almost zero by the end of May. Few cases were reported during May through September. The AH scatterplot shows periodic change: the greatest increase was in August and greatest decrease was in February. In the time-series analysis, we excluded data from May 1 through September 30 in each year and observed a total of 544 days in our sample. The mean (SD) number of influenza cases in the sample was 5.79 (2.64), and mean (SD) AH was 176.06 (126.26).

**Figure 2:**
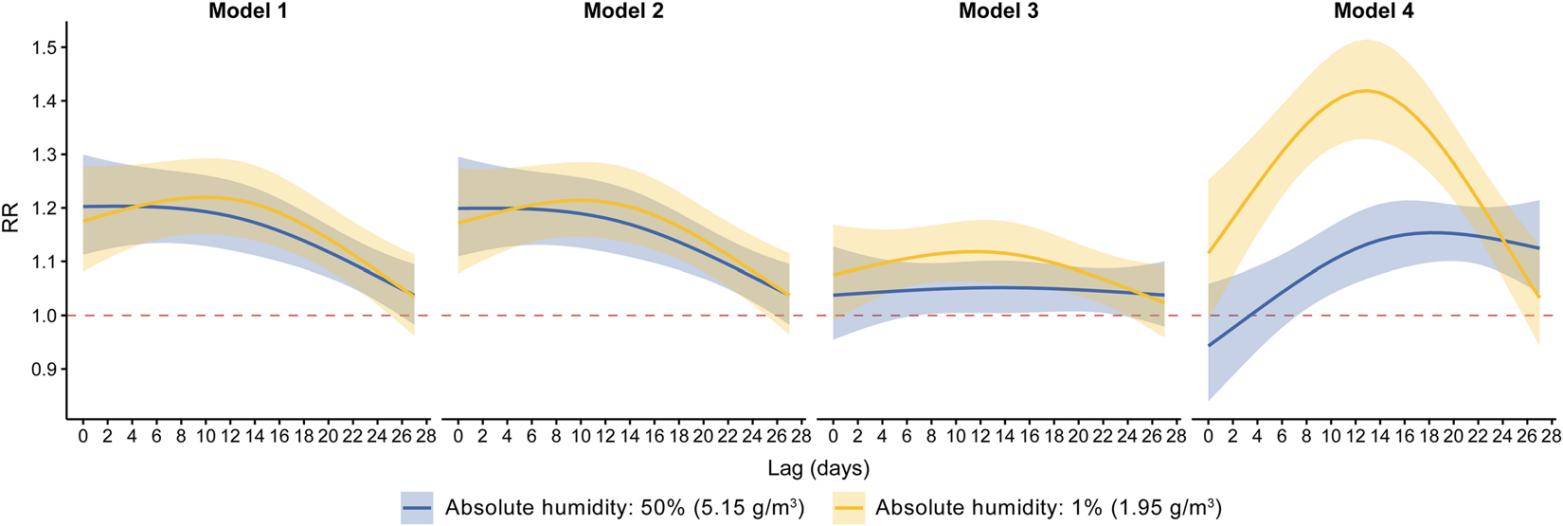
Lag-response relationship at lag 50th (blue) and 1st (yellow) percentile of AH by four models. Solid line and shaded area represent relative risk (RR) and its 95% confidence interval respectively. Horizontal dotted line in red show RR = 1 representing no difference in risk.

Figure 2 shows the RR-lag relationship for the 4 models at 50th and 1st percentiles of AH. RR (relative risk) refers to the multiple of influenza cases at a given AH percentile, as compared with the reference value-the 95th percentile of AH. In all models, a curve with a clear peak and inverted U-shape appears at the 1st percentile of AH. The RR approaches its maximum at lag 10–14 days. A comparison of the models showed that Models 1 and 2 had almost identical graphs and associations. At 1st percentile of AH, model 4 had the highest peak RR, 1.42 (95% CI, 1.33–1.51), while Model 3 had a moderate RR, 1.12 (95% CI, 1.06–1.18).

**Figure 3:**
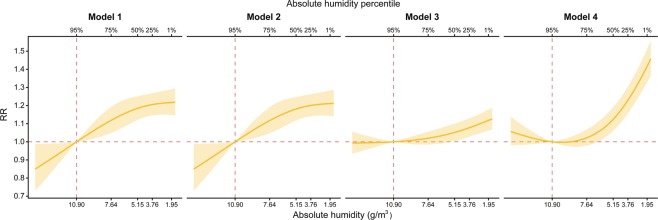
Exposure-response relationship at lag 12 by four models. Yellow solid line and shaded area represent relative risk (RR) and its 95% confidence interval respectively. Horizontal and vertical dotted lines in red show reference value of AH and RR = 1 representing no difference in risk.

Figure 3 shows the exposure-response relationship for the 4 models at lag 12. In all models, RR increased as AH decreased. A comparison of the models showed, again, that Models 1 and 2 yielded almost identical associations. As was the case for the RR-lag relationship (Fig. 2), at lag 12 and a 1st percentile of AH, Model 4 had the highest RR and Model 3 had the lowest RR among four models.

**Figure 4:**
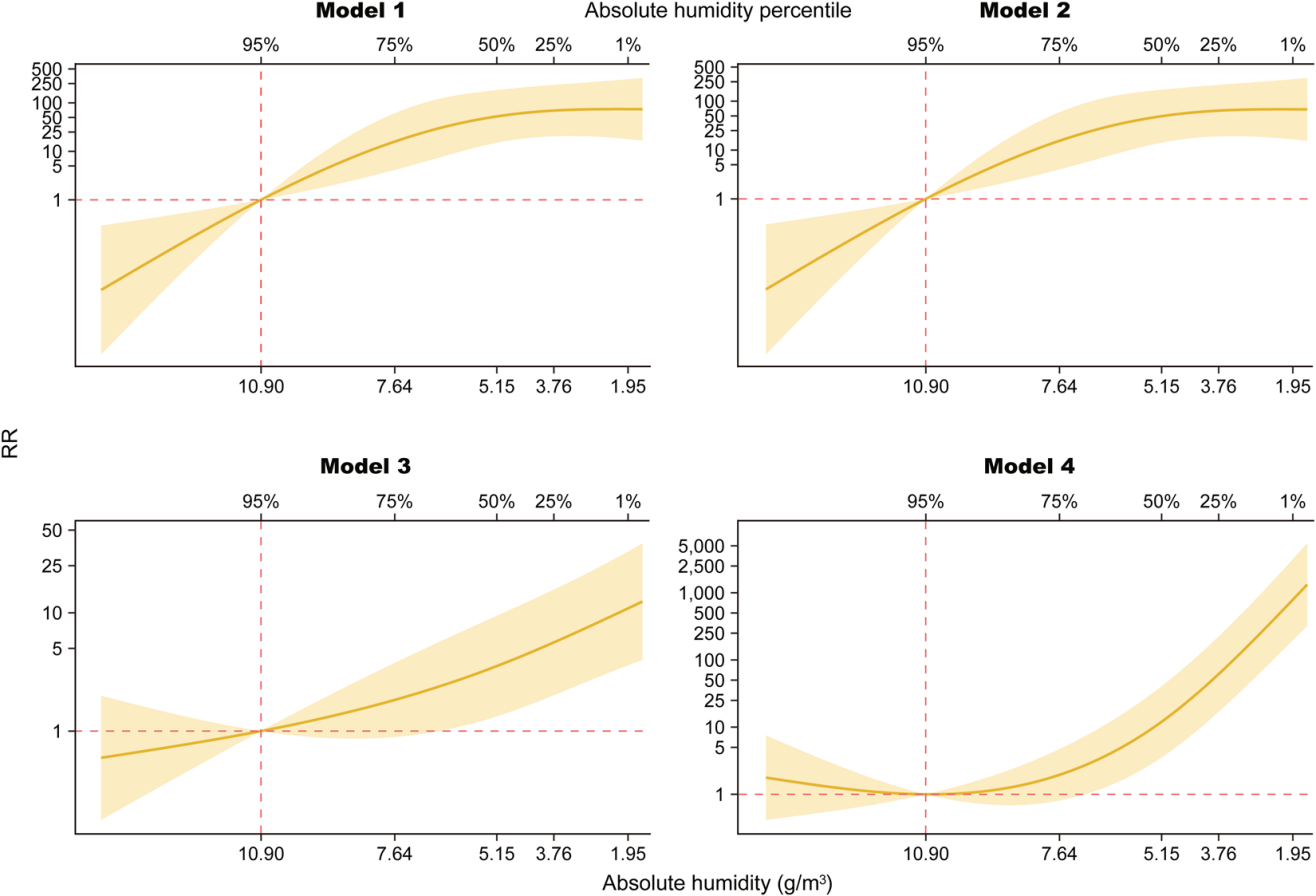
Overall cumulative exposure-response relationship by four models. Yellow solid line and shaded area represent relative risk (RR) and its 95% confidence interval respectively. Horizontal and vertical dotted lines in red show reference value of AH and RR = 1 representing no difference in risk. Scale size in vertical axis is transformed into common logarithm.

Figure 4 shows the overall effects of AH on influenza cases, including all delayed effects up to lag27. In all models, RR increased as AH decreased. A comparison of the models revealed 3 key findings. First, Models 1 and 2 yielded almost identical associations. Second, RRs increased logarithmically in Models 1 and 2 and exponentially in Models 3 and 4. Third, the magnitude of RR estimates at the 1st percentile of AH differed largely between Models 1/2, 3, and 4. The respective values were 74.2 (95% CI, 18.0–306.4), 10.9 (95% CI, 3.6–32.9), and 789.2 (95% CI, 198.1–3142.8) (See Supplementary Information).

## Discussion

This study analyzed daily influenza surveillance data with distributed lag non-linear model to investigate associations of AH with influenza and the strength of associations. To the author’s knowledge, this is the first study to compare different adjustment for seasonality and autocorrelation in regression model. The results for all 4 models clearly show a significant increase in the number of influenza cases as AH decreased, although the magnitude of the association differed substantially by model. The lag between extremely low AH (1st percentile) and the greatest number of persons diagnosed at clinics was 10–14 days.

The present findings are consistent with those of previous laboratory studies^12,13^, which showed a high correlation between influenza virus survival and AH. Past and present evidence suggests that increased survival of influenza virus at low AH increases virus transmission and the number of infected persons. In addition, our results are in line with previous epidemiological study^14^, which analyzed weekly surveillance data in Hong Kong, confirming the negative association of AH with influenza infection both in temperate climate and subtropical climate. However, comparison of the strength of present and past associations is difficult because the reference values used in prior studies using weekly data differed from those used in this study. In addition, no previous study reported lag for peak RR at low AH, so direct comparison is not possible. Although the present results showing a lag of 10–14 days from the day of low AH to the day of diagnosis were consistent among models, a previous study estimated that the incubation period for influenza was 1.5–3 days^30–32^. Future studies should attempt to resolve this discrepancy by analyzing daily data from other areas.

The objective of the article is to compare and discuss the results from the 4 different models rather than to choose one best model. First, the results of Models 1 and 2 were almost identical, which indicates that variation in the number of influenza cases can be adequately explained with all variables except for the term of controlling autocorrelation. The results of Models 3 and 4 differed; thus, the smoothing splines used to control for seasonality in Model 1 and 2, ns(date), have largely accounted for the autocorrelation term. Second, the RR scale was substantially lower when the autocorrelation term (the logarithm of lagged outcome counts, log(*Y*_*t*−1_)) was incorporated into the model (Model 3 v.s. 4). This suggests that a large proportion of cases occurring at time *t* was dependent on the size of the infected population, i.e. the number of cases in the recent past, and that inclusion of past cases caused a downward bias in the total effect of AH exposure, if we assume that part of the effect was attributable to a causal path involving the more immediate impact of that exposure, which caused higher counts and more infected persons at time *t* − 1^33^. Third, the seasonality control affected the RR differently depending on the autocorrelation control. The RR decreased in the model with autocorrelation by changing seasonality control function from ns(date) to ns(epidemic year) + ns(day of epidemic year), i.e. from Model 1 to Model 3. On the other hand, the RR increased in the model without autocorrelation by the switch of seasonality control, i.e. from Model 2 to Model 4. Thus, future studies analyzing daily influenza data should recognize how different specifications for seasonality control yield different results.

Daily rather than weekly influenza data were analyzed, and this granularity of data is a strength of this study. Influenza surveillance data are frequently available only on a weekly basis; daily data are not easy to obtain. Although daily meteorological data are available, most previous studies analyzed weekly influenza data and thus converted daily meteorological data to weekly averages. Obviously, such conversion results in loss of information on small daily changes in meteorological factors. Thus, the availability of daily data increases granularity. Another strength of this study is its comparison of approaches to adjusting for seasonality and autocorrelation. We showed that varied approaches indicated presense of associations, although the strength of the associations varied greatly between models. These findings might assist in the development of models in future research.

Our study had several limitations. First, the misclassification of the outcome, influenza cases, might occur. Although the real-time surveillance system requests all city medical facilities to report influenza cases, reporting is not mandatory; therefore, data are missing for some influenza cases. If nonreporting of influenza cases is independent of AH, misclassification would be nondifferential and might lead to underestimation of the association. However, the nature and severity of any bias cannot be accurately determined in the current system, because we have no means to ascertain whether the absence of an observation of an influenza case was due to the lack of a diagnosis or to a reporting failure. Second, the potential measurement errors in the exposure, meteorological factors, might be another limitation. Data on relative humidity and temperature, which are used for calculating AH, were collected outdoors in the city. However, during winter - the influenza season - most people spend more time indoors than outdoors. Thus, the present AH values might not represent actual exposures. Nonetheless, a previous study reported that indoor AH was strongly correlated with outdoor AH (*ρ* = 0.96)^34^. Thus, any bias resulting from nondifferential misclassification is likely to be limited. Third, the study results were based on the data from only one area, Kawasaki city. The models should be verified with a dataset from different area. Fourth, the lack of other meteorological factors in our regression model, which previous studies reported as factors associated with influenza infection, is another limitation. Temperature was excluded due to strong collinearity with AH (*ρ* = 0.93, *p* = 0.01) to avoid multicollinearity. Solar radiation was not included due to unavailability of data in Kawasaki city. Future studies should deal with this issue by incorporating these factors into the model.

## Methods

This study was approved by the ethics committees of School of Medicine, Toho University (Approval No.A17022), located in Tokyo, Japan.

### Study area and characteristics

**Figure 5:**
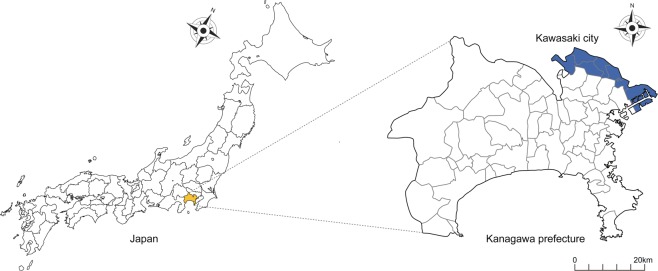
Map of Kanagawa prefecture and Kawasaki city.

Kawasaki City was selected as the study area. The city is located in northeast Kanagawa Prefecture, which is adjacent to Tokyo (Fig. 5). It is divided into 7 administrative districts and has an area of 144.35 km^2^. The population was 1,503,690 in 2017, making it the seventh largest city in Japan. Kawasaki has a temperate climate with 4 distinct seasons; it is hot and humid in summer and cold and dry in winter.

The Kawasaki City Infectious Disease Surveillance System collects real-time data on influenza infections^35^. Since March 2014, the system has required all city medical facilities to report the number of daily influenza diagnoses, by sex, age category, and area. Rapid diagnosis kits are used to differentiate influenza types A and B, and surveillance data are available from a city-run website (See Data Availability Statement). For this study, data on the daily total numbers of influenza A cases were obtained.

Daily meteorological data were obtained from a website providing information on air quality in Kawasaki, as were meteorological observational data, such as hourly temperature and hourly relative humidity, in each of the 7 city districts. These data were used to calculate hourly AH in each district, according to the following formula^36^:

1 $$AH(g/{m}^{3})=\frac{VP\times 216.5}{273.15+T}$$

where T is temperature and VP is vapor pressure, which is used to define relative humidity (RH) as

2 $$VP(hPa)=6.11\times {10}^{\frac{7.59\times T}{T+240.72}}\times \frac{RH}{100}.$$

Average hourly AH was then calculated in all 7 districts, after which average daily values were obtained.

### Statistical analysis

Time-series analysis was used to examine the association between daily AH and number of influenza cases. Quasi-Poisson regression was used to address overdispersion in count data^22,33,37–39^. To investigate the nonlinear and delayed effects of AH on the daily number of influenza cases, we used a distributed lag nonlinear model^40^, expressed as

3 $$\begin{array}{rcl}{Y}_{t} & \sim  & {\rm{Poisson}}({\mu }_{t})\\ \log ({\mu }_{t}) & = & \alpha +{\rm{cb}}({\rm{AH}})+\beta \cdot {{\rm{DOW}}}_{t}+\gamma \cdot {{\rm{holiday}}}_{t}+{\rm{offset}}(\log ({\rm{clinic}}))\\  &  & +\,f({\rm{seasonality}})+g({\rm{autocorrelation}})\end{array}$$

where subscript *t* is the day of observation, *Y*_*t*_ is the observed number of influenza cases on day *t*, and *α* is the intercept. The main exposure, AH, is transformed by function *cb*(·)-a cross-basis function to represent the 2-dimensional relationship between exposure and its lagged effects; natural cubic splines were used for exposure response and lag response. Two control variables were used: DOW_*t*_ is a vector for dummy variables representing the day of the week on day *t*, and holiday_*t*_ is a dummy variable that takes the value 1 if day *t* is a holiday. An offset term for the total number of medical facilities by day, offset(log(clinic)), is added to adjust for daily variation in the number of medical facilities reporting influenza cases.

We investigated how adjustments for seasonality and autocorrelation might affect the estimation of associations. Although many previous studies used a single natural cubic spline of the date for seasonal adjustment, some recent studies have attempted to use decomposed seasonality terms, i.e. natural cubic splines of the epidemic year and day of epidemic year^41^. Some studies using autocorrelation adjustment reported that adding an autoregressive term increased goodness of fit, but others found that including such past cases in the model could cause a downward bias because past cases are an intermediate variable between past exposure and present cases^42,43^. Thus, on the basis of these previous findings, we constructed 4 models with 2 patterns for each adjustment, represented by *f*(seasonality) and *g*(autocorrelation), as follows:

- Model 1: *f*(seasonality) = *ns*(date, df = 4/year), *g*(autocorrelation) = log(*Y*_*t*−1_)
- Model 2: *f*(seasonality) = *ns*(date, df = 4/year), *g*(autocorrelation) = 0
- Model 3: *f*(seasonality) = *ns*(epidemic year, df = 2) + *ns*(day of epidemic year, df = 2), *g*(autocorrelation) = log(*Y*_*t*−1_)
- Model 4: *f*(seasonality) = *ns*(epidemic year, df = 2) + *ns*(day of epidemic year, df = 2), *g*(autocorrelation) = 0

where epidemic year indicates the period from October 1 to April 30, and *ns* indicates natural cubic spline.

This analysis has other potential confounders. Temperature and solar radiation were reported as confounders in a previous study^44–46^. However, because temperature was highly correlated with AH and solar radiation was not available from the website, we excluded them from our model.

Maximum lag was set to 28 days in the model. The degrees of freedom in the natural cubic spline for *cb*(·), *f*(·) and *g*(·) were determined by using Akaike’s Information Criterion for quasi-Poisson (Q-AIC) values in a grid search. An excess number of outcome zeros could potentially bias estimated values. Therefore, we removed data from May 1 to September 30, during which there was no epidemic of type A Influenza. The period of the regression analysis was thus October 1, 2014 to April 30, 2017. To assess associations, relative risks (RRs) and their confidence intervals (CIs) were estimated, and the 95th percentile of AH in the above period was used as the reference value. The analysis was performed by using the *dlnm* package in R, version 3.5.2.

## Supplementary information

Supplementary information

Supplementary information 2.

Supplementary information 3.

## Data Availability

The surveillance data and meteorological data analysed during the current study are available in the website repository, https://kidss.city.kawasaki.jp/en/modules/realsurveillance/ and http://sc.city.kawasaki.jp/taiki/HOURLY/HT01I20190509.html.
