# Supplementary material for: Association Between Seasonal Influenza and Absolute Humidity: Time-Series Analysis with Daily Surveillance Data in Japan
Source: Sci Rep. 2020 May 8;10:7764. doi: 10.1038/s41598-020-63712-2 (PMC7211015; doi:10.1038/s41598-020-63712-2)
Supplement: Supplementary file 1 — Supplementary information [file 41598_2020_63712_MOESM1_ESM.pdf]

Supplementary Information

Title: Association Between Seasonal Influenza and Absolute Humidity: Time-Series Analysis with Daily Surveillance Data in Japan

Keita Shimmei[1,2,*], Takahiro Nakamura[2], Chris Fook Sheng Ng[3], Masahiro Hashizume[4], Yoshitaka Murakami[5], Aya Maruyama[6], Takako Misaki[6], Nobuhiko Okabe[6], and Yuji Nishiwaki[2]

[1]The World Bank, The Poverty and Equity Global Practice, Washington D.C., U.S.
[2]Toho University, Department of Environmental and Occupational Health, Tokyo, Japan
[3]Nagasaki University, School of Tropical Medicine and Global Health, Nagasaki, Japan
[4]Nagasaki University, Department of Paediatric Infectious Disease, Nagasaki, Japan
[5]Toho University, Department of Medical Statistics, Tokyo, Japan
[6]Kawasaki City Institute for Public Health, Kawasaki, Japan
[*]shinmeikeita@gmail.com

Table S1. Relative risk and 95% confidence interval by models, by lags at 6, 12, 18 and 24, and by AH percentiles at 75%, 50%, 25% and 1%.

Lag	Percentile	AH	Model 1			Model 2			Model 3			Model 4		
			RR	95% CI		RR	95% CI		RR	95% CI		RR	95% CI	
				low	high		low	high		low	high		low	high
6	75%	7.64	1.16	1.09	1.23	1.15	1.08	1.23	1.02	0.98	1.06	0.97	0.92	1.02
	50%	5.15	1.20	1.13	1.27	1.20	1.13	1.27	1.05	1.00	1.10	1.04	0.98	1.11
	25%	3.76	1.21	1.14	1.27	1.20	1.14	1.27	1.07	1.02	1.12	1.13	1.06	1.21
	1%	1.95	1.21	1.14	1.28	1.21	1.14	1.28	1.11	1.05	1.16	1.30	1.22	1.39
12	75%	7.64	1.12	1.05	1.19	1.12	1.05	1.19	1.02	0.98	1.06	1.02	0.98	1.07
	50%	5.15	1.18	1.12	1.25	1.18	1.12	1.25	1.05	1.00	1.10	1.12	1.06	1.19
	25%	3.76	1.21	1.15	1.27	1.20	1.14	1.26	1.08	1.03	1.13	1.23	1.16	1.30
	1%	1.95	1.22	1.15	1.29	1.21	1.14	1.28	1.12	1.06	1.18	1.42	1.33	1.51
18	75%	7.64	1.08	1.02	1.14	1.08	1.02	1.14	1.02	0.99	1.06	1.06	1.03	1.10
	50%	5.15	1.14	1.09	1.19	1.14	1.08	1.19	1.05	1.01	1.09	1.15	1.10	1.21
	25%	3.76	1.16	1.11	1.21	1.16	1.11	1.21	1.07	1.02	1.12	1.23	1.16	1.29
	1%	1.95	1.17	1.11	1.23	1.17	1.10	1.23	1.10	1.05	1.15	1.34	1.27	1.42
24	75%	7.64	1.03	0.98	1.08	1.03	0.99	1.08	1.03	0.99	1.06	1.10	1.05	1.14
	50%	5.15	1.07	1.03	1.12	1.07	1.03	1.12	1.04	1.00	1.09	1.14	1.08	1.20
	25%	3.76	1.09	1.04	1.13	1.09	1.04	1.13	1.05	1.00	1.10	1.15	1.08	1.21
	1%	1.95	1.08	1.02	1.15	1.08	1.02	1.15	1.05	1.00	1.10	1.14	1.07	1.22

Table S2. Accumulation of Relative risk over all lag days by models and by AH percentiles at 75%, 50%, 25% and 1%.

Percentile	AH	Model 1			Model 2			Model 3			Model 4		
		RR	95% CI		RR	95% CI		RR	95% CI		RR	95% CI	
			low	high		low	high		low	high		low	high
75%	7.64	15.9	4.2	60.8	15.5	4.0	59.3	1.8	0.9	3.8	1.9	0.8	4.7
50%	5.15	52.5	15.1	182.8	49.3	14.2	171.3	3.5	1.3	9.4	12.0	3.6	40.5
25%	3.76	69.3	20.5	234.1	64.1	19.1	215.5	5.6	2.0	15.7	61.5	17.0	222.9
1%	1.95	74.2	18.0	306.4	68.3	16.6	281.0	10.9	3.6	32.9	789.2	198.1	3143.8
